# Extending the CRISPR toolbox for *C. elegans: Cpf1* as an alternative gene editing system for AT-rich sequences

**DOI:** 10.17912/W2237D

**Published:** 2017-12-01

**Authors:** Annabel Ebbing, Peng Shang, Niels Geijsen, Hendrik Korswagen

**Affiliations:** 1 Hubrecht Institute, Royal Academy of Arts and Sciences and University Medical Center Utrecht, Uppsalalaan 8, 3584 CT Utrecht, The Netherlands; 2 Department of Clinical Sciences of Companion Animals, Faculty of Veterinary Medicine, Utrecht University, Yalelaan 108, 3584 CM Utrecht, the Netherlands

**Figure 1.**
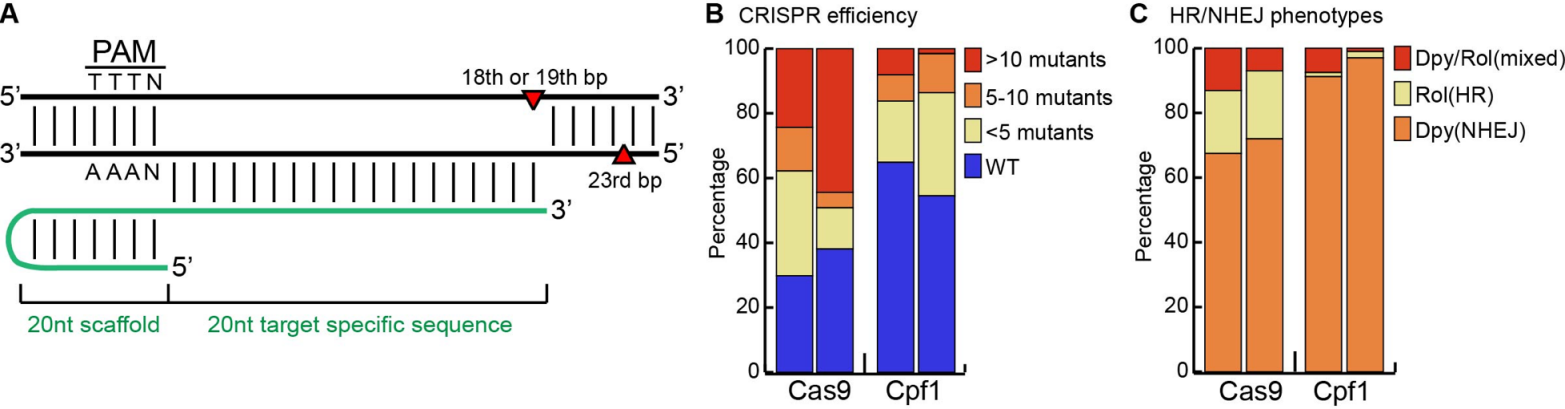
(A) Schematic overview of the Cpf1 crRNA bound to its genomic target sequence. The staggered cut-site is indicated in red. (B) Percentage of Cas9 or Cpf1 injected animals segregating a Dpy or Rol phenotype (n=37, 63, 37 and 66). (C) Percentage of Dpy (loss of function), Rol (*cn64* mutation) or DpyRol (*cn64*/null or homozygous null) among mutant phenotypes.

## Description

The CRISPR/Cas9 system has become a powerful tool for genome-editing in *C. elegans*. Sequence specificity of the system is determined by a guide RNA which targets the Cas9 endonuclease to the genomic region of interest. In addition, Cas9 needs to interact with a so-called protospacer-adjacent motif (PAM) next to the target sequence. In case of Cas9 from *Streptococcus pyogenes* (SpCas9), the most commonly used Cas9, this sequence is NGG.

Because the *C. elegans* genome sequence is relatively AT-rich, especially in non-coding regions such as introns and promoters (*C. elegans* sequencing consortium, 1998), it is not always possible to find an appropriate NGG PAM motif to target the genomic region of interest. For such cases, it would be helpful to have an alternative CRISPR system that is compatible with AT-rich sequences.

Recently, another group of single-effector CRISPR/Cas proteins, Cpf1, has been identified that displays a preference for T-rich PAM motifs (Zetsche et al., 2015). Cpf1 from *Acidaminococcus* sp. BV3L6 (AsCpf1), recognizes the PAM motif (T)TTN and shows robust nuclease activity in human cells (Zetsche et al., 2015). In contrast to Cas9, Cpf1 does not create a blunt-ended double strand break, but forms a staggered cut with a 5’ overhang (Fig. 1A) (Zetsche et al., 2015). Furthermore, Cpf1 does not require a separate tracrRNA. A single 44nt crRNA is sufficient and this crRNA can be further shortened to 40nt without loss of efficiency (Yamano et al., 2016). Here, we show that CRISPR/Cpf1 can also efficiently mediate genome editing in *C. elegans*, providing a valuable addition to the CRISPR toolbox that is available for this system.

We used the well-established *dpy-10* system to simultaneously assay the frequency and mechanism of repair of the Cpf1-induced double strand break (Arribere et al., 2014; Paix et al., 2015). Injection of recombinant AsCpf1 protein with a *dpy-10* crRNA and a single-stranded DNA oligo repair template (ssODN) containing the dominant *cn64* mutation resulted in the formation of Rol (homologous recombination, HR, with insertion of the *cn64*mutation) and Dpy (non-homologous end-joining, NHEJ, with loss of function resulting from deletion or insertion of sequences during the repair process) progeny. Using this assay, we compared the HR and NHEJ mediated repair frequencies between SpCas9 and AsCpf1 injected animals. In two independent experiments, 35% and 45% of the AsCpf1 injected animals produced progeny with a Dpy or Rol phenotype (Fig. 1B). Of these, 54% and 70% showed less than 5 progeny with a Dpy or Rol phenotype, while 23% and 3% showed >10 Dpy or Rol progeny per injected animal (so-called “jackpot” plates).

Parallel experiments with purified SpCas9 protein produced similar results, although in both cases the number of injected animals segregating Dpy or Rol phenotypes (70% and 62%) and the percentage of jackpot plates (35% and 72%) was higher. Furthermore, although both Cas9 and Cpf1 induced the formation of Dpy and Rol progeny, the percentage of Rol progeny was lower in both Cpf1 experiments (Fig. 1C). Whether this reflects a difference in endonuclease activity of Cas9 and Cpf1, a difference between the *dpy-10* crRNAs, cut sites, and repair template requirements, or a difference in repair mechanisms remains to be established.

Taken together, these results demonstrate that Cpf1 is active in *C. elegans* and that the *dpy-10* locus can be used as a co-conversion marker for Cpf1 mediated genome-editing.

## Reagents

Recombinant Cas9 and Cpf1 protein: Recombinant SpCas9 or AsCpf1 protein were purified from *E. coli* (BL21(DE3)) as described (D’Astolfo et al., 2015). Limited quantities can be requested from the Utrecht University Gene Editing Facility (genedit@uu.nl), but can also be obtained from commercial sources.

Cpf1 specific crRNA for *dpy-10*: The crRNA consists of the AsCpf1 scaffold sequence (UAAUUUCUACUCUUGUAGAU) and a 20 nt guide RNA sequence. To find appropriate guide RNAs, the genomic sequence surrounding the location of the *cn64* mutation was manually searched for (T)TTN PAM motifs and 20 nt sequences were selected and tested for potential off-target binding using CRISPR RGEN tools (http://www.rgenome.net/cas-offinder/). The final crRNA for *dpy-10* was UAAUUUCUACUCUUGUAGAU + CAGUCAUUCUCAUCUUGCCG.

Single-stranded oligo-DNA repair template for generating *dpy-10(cn64)*: A single-stranded oligo-DNA (ssODN) repair template was designed that can be used for both Cpf1 and Cas9 mediated genome-editing of the *dpy-10* locus. The sequence of the repair template is: AGATCAAA CGACGAGGCTGCACTTGAACTTCAATACGGCAAGATGAGAATGACTGGGAACCGTACAGCATGCGGTGCCTATGGTAGCGGAGCATCACATGGCT TCAGACCAACAGCCTATGGAGATGAAATCACTGGAGCTCCA (synthetized by IDT as a 4 nM ultramer, salt free and diluted to 500 ng/μl working stock in nuclease free water). Yellow: silent point mutation (A to G) to disrupt the Cpf1 PAM motif. Green: silent point mutation (C to A) to disrupt the Cas9 PAM motif. Red: *cn64* mutation (TCGT to ATGC).

Injection mixture: The injection mix (prepared fresh before injection as described in Paix et al., 2015): Cas9: Recombinant SpCas9 protein (75 μM stock solution): 1.2 μl*dpy****‐****10* crRNA from Paix et al. (4.0 μg/μl (IDT, 100 nM oligo, standard desalting): 0.8 μl tracrRNA (5.0 μg/μl (IDT, 100nM oligo, HPLC purified): 2.0 μl*dpy****‐****10* ssODN repair template (500 ng/μl (IDT, 4nM ultramer, standard desalting): 0.47 μl Nuclease-free water to reach a final volume of 10 μl (no salts or buffer needs to be added)

Cpf1: Recombinant AsCpf1 protein (75 μM stock solution): 1.2 μl*dpy****‐****10* crRNA (4.0 μg/μl (IDT, 100 nM oligo, standard desalting): 0.8 μl*dpy****‐****10* ssODN repair template (500 ng/μl (IDT, 4nM ultramer, standard desalting): 0.47 μl Nuclease-free water to reach a final volume of 10 μl (no salts or buffer needs to be added)

The readout for homologous recombination of the *cn64* mutation (Rol) and non-homologous end-joining resulting in loss of function (Dpy) was as described (Arribere et al., 2014).
